# Motorcycle-Related Traumatic Brain Injuries: Helmet Use and Treatment Outcome

**DOI:** 10.1155/2015/696787

**Published:** 2015-03-23

**Authors:** Mathias Ogbonna Nnanna Nnadi, Olufemi Babatola Bankole, Beleudanyo Gbalipre Fente

**Affiliations:** ^1^Division of Neurosurgery, Department of Surgery, University of Calabar Teaching Hospital, Calabar, Nigeria; ^2^Neurosurgical Unit, Department of Surgery, Lagos University Teaching Hospital, Idi-Araba, Lagos, Nigeria; ^3^General Surgery Unit, Department of Surgery, Niger Delta University Teaching Hospital, Okolobiri, Bayelsa State, Nigeria

## Abstract

*Summary*. With increasing use of motorcycle as means of transport in developing countries, traumatic brain injuries from motorcycle crashes have been increasing. The only single gadget that protects riders from traumatic brain injury is crash helmet. *Objective*. The objectives were to determine the treatment outcome among traumatic brain injury patients from motorcycle crashes and the rate of helmet use among them. *Methods*. It was a prospective, cross-sectional study of motorcycle-related traumatic brain injury patients managed in our center from 2010 to 2014. Patients were managed using our unit protocol for traumatic brain injuries. Data for the study were collected in accident and emergency, intensive care unit, wards, and outpatient clinic. The data were analyzed using Environmental Performance Index (EPI) info 7 software. *Results*. Ninety-six patients were studied. There were 87 males. Drivers were 65. Only one patient wore helmet. Majority of them were between 20 and 40 years. Fifty-three patients had mild head injuries. Favorable outcome among them was 84.35% while mortality was 12.5%. Severity of the injury affected the outcome significantly. *Conclusion*. Our study showed that the helmet use by motorcycle riders was close to zero despite the existing laws making its use compulsory in Nigeria. The outcome was related to severity of injuries.

## 1. Introduction

The increasing use of motorcycles in commercial transportation in developing countries has led to explosion of their number in many countries [1–3]. In Nigeria, the trend is the same [4, 5]. Increasing number of motorcycles was associated with increasing number of motorcycle crashes [1]. Per vehicle mile travelled, motorcycle riders have a 34-fold higher risk of death in a crash than people driving other vehicles and are 8 times more likely to be injured [6]. The use of helmet reduced head injuries among riders [7]. We studied motorcycle-related traumatic brain injury patients in our center over a three-and-half-year period.

## 2. Methods

It was a prospective, descriptive, and cross-sectional study involving motorcycle-related traumatic brain injury patients managed in our center from August 2010 to January 2014. Patients were managed using our protocol for traumatic brain injuries.

### 2.1. Our Protocol

Patients were managed in accident and emergency using advanced trauma life support (ATLS) protocols (primary and secondary surveys). In primary surveys, patients were resuscitated ensuring patent airways and oxygen saturation of 95% and above. We used normal saline to maintain blood volume aiming at euvolemia and normotension. We gave adequate analgesia, antiepileptic drugs in posttraumatic seizures, and calm aggressive patients with chlorpromazine. Quick checks for other organ injuries that could be life threatening to patients were made. In secondary survey, we took detailed history and physical examination of patients. Glasgow Coma Scores after resuscitation of patients were assessed. Appropriate investigations were carried out based on needs and their affordability. Patients with severe traumatic brain injuries were admitted to intensive care unit while those with mild and moderate traumatic brain injuries were admitted to the wards. Patients with CT scan lesions not requiring surgery and those who could not afford CT scan were managed nonoperatively. Patients with lesions requiring surgery such as extradural, subdural, and intracerebral hematomas/contusions and depressed skull fractures had surgical care. Surgical procedures included craniotomy for acute extradural, acute subdural, and intracerebral hematomas/contusions, burr hole for subacute and chronic subdural hematomas, and craniectomy with primary bone fragment replacement or depressed bone elevation for depressed skull fractures. Associated injuries were managed by appropriate specialist units.

We gave broad spectrum antibiotics (to those who had open tissue injuries), multivitamins, high energy and high protein diet. The diet was constituted thus: 500 mL pap, two tablespoonsful of powdered milk, one tablespoonful of red oil, two tablespoonsful of soya bean powder, and one tablespoonful of crayfish powder. The diet was given five to six times daily via nasogastric tubes or orally. Their daily fluid requirements were calculated and factored into the fluid content of the diet. We used locally prepared diet because most of our patients could not afford Complan or Casilan and there was no functional dietetic unit in our hospital. On discharge, patients were followed up in the outpatient clinic and by phone calls when they failed to attend the clinic.

Data were collected using structured proforma which was part of our prospective data bank that was approved by our hospital's ethics and research committee. The biodata, position of patient at the time of crash (driver, passenger, or pedestrian), helmeted or not helmeted at the time of crash, severity of injury (using Glasgow Coma Score after resuscitation), and other clinical signs were collected in accident and emergency (A&E) unit. Radiological findings were documented either in A&E or in the wards depending on the time the radiological imaging was done. The progress of the patients was documented in the wards. The functional outcome was determined using Glasgow Outcome Score (GOS) [8]. It classifies patients into 1 dead, 2 vegetative state, 3 severe disability, 4 moderate disability, and 5 good recovery. Four and five are regarded as good (favorable) functional outcome. The functional outcome was determined in outpatient clinic or by phone discussion (those who failed to attend the clinic) at three months after injury. The functional outcome three months after injury had been found to be the best predictor in the long term [9].

Motorcycle drivers, their passengers, and pedestrian hit by motorcycles were included in the study. Occupants of vehicles involved in crashes with motorcycles were excluded from the study.

### 2.2. Statistical Analysis

Data were analyzed using Environmental Performance Index (EPI) info 7 software (Center for Disease Control and Prevention, Atlanta, Georgia, USA: http://wwwn.cdc.gov/epiinfo/7/index.htm).

The visual band package was used for the analysis. The “frequency gadget” was used to analyze gender, helmet use, severity of injury, and mode of treatment. We used the “mean gadget” to determine the mean age of the patients. The ages were recoded in groups of tens and their frequency was determined also. “MxN/2X2 table” was used in analyzing two variables such as effect of severity of injury on outcome, while its advanced part was used in three variables such as position of patient, severity of injury, and outcome. With 95% confidence interval, *P* < 0.05 was considered significant.

## 3. Result

There were 96 patients in the study. Males were 87 (90.32%) while females were nine (9.68%). Their ages ranged from three years to 69 years with mean age of 31.89 years. Majority of patients (56.25%) were 20–40 years (Table 1).

There were 65 motorcycle drivers, 22 passengers, and nine pedestrians. Of the 87 drivers/passengers 98.85% (86) did not wear helmet at the time of crash while 1.15% (one patient) wore helmet. Fifty-three patients had mild injuries, 29 had moderate injuries, and 23 had severe head injuries. Forty-four patients (45.83%) did CT scan. Thirty-eight of them (86.36%) had intracranial lesions while six patients had no intracranial lesion. There were multiple lesions in 18 patients' CT scans (Table 2). Eighty-one patients were managed nonoperatively, while 15 patients had surgical care. Three patients had burr holes for subacute/chronic subdural hematomas, six had craniotomy for acute subdural/epidural hematoma, three had craniectomy with bone fragments replacement for depressed skull fractures, and three had repair of scalp avulsions.

The overall favorable functional outcome was 84.38% and mortality was 12.5%. Favorable outcome among those with severe head injury was 52.17%. Severity of the injury significantly affected the outcome, *P* = 0.0002 (Table 3). Presence of intracranial lesions did not significantly affect the outcome, *P* = 0.6178.

## 4. Discussion

Ninety-six patients were studied and males formed 90.32% (87). The majority of patients were between 20 and 40 years. They were young men in their prime trying to make ends meet through commercial motorcycle driving since collar jobs were hard to come by in our country. With long traffic holdups in our city, many commuters use motorcycles as ways of circumventing the holdups. The patronage encourages many young ones to join commercial motorcycle driving as source of living. These had been documented by many authors [10–17]. Low percentage of patients who could afford CT scan led credence to the level of income among the patients.

Of 86 drivers/passengers, only one patient (1.16%) wore helmet. Nwadiaro et al. [14] in their study in north-central Nigeria found 100% not using helmet. Arosanyin et al. [18] in Zaria, Nigeria, found 16% helmet use in their study. In Ilorin, Nigeria, Arosanyin [19] found 13.5% helmet use. The reasons they found were cost of acquisition and weak enforcement of helmet laws. In Iribhogbe and Odai [20] study, they found that many motorcyclists complain of cost of helmet while many had helmets but refused to wear them due to “inconveniencies.” In spite of overwhelming evidence that helmet use prevented head trauma [7, 21], there had been hostilities towards helmet use mainly due to cost, ignorance, drug use, and discomfort [10]. “In United States of America (USA) similar hostilities had been documented. In 1966, the congress mandated US Department of Transportation to withhold federal funds from states without mandatory helmet laws. In 1975 organization such as ABATE (A Brotherhood Against Totality Enactment) lobbied USA congress to repeal the mandatory helmet laws. Their reasons included reduced visibility, reduced hearing, and neck injuries. These were not validated by scientific evidence. Repeal of the law by the congress led many states to repeal their own [22].” An evidence based review of articles from 1990 to 2009 by Macleod et al. [23] in USA showed that the use of helmets decreased the overall death rate of motorcycle crashes when comparing helmeted with nonhelmeted patients; helmet decreased the incidence of lethal head injury in crashes compared to nonhelmeted ones; helmet decreased the severity of nonlethal head injury compared to nonhelmeted ones; helmet laws reduced mortality and head injuries in areas with the law compared with those without the law. The protective effect of helmet use was also found in the study across three cities in Europe (Hannover, Munich, and Glasgow) by Richter et al. [12]. In Italy, Latorre et al. [24] studied 736 injured riders and found 12% helmet use but the protective effect of helmet was significant. In Jamaica, opponents of helmet law said that crashes in developed countries were due to high speedways and well maintained roads unlike windy poor surfaced and congested local highways in their country where the speed was low. They claimed that the injuries in their roads would be less severe. Study carried out by Crandon et al. [10] in University of West Indies showed otherwise. In many developing countries high nonhelmet use had been documented by many authors [1, 25–27]. These showed that low level of helmet use was not peculiar to our center but a global “disease.” The fact that the only helmeted patient in our study had severe head injury was of note. Head injury had been found to be the leading cause of death even in helmeted riders [28]. It had also been deduced that helmet and the other safety equipment showed efficacy in reducing deaths or serious injuries, but they had not been sufficient for safe lives [29, 30]. It should be noted that obedience to traffic laws is complimentary to helmet use. The failure of drivers to comply with basic road safety legislation was the main cause of serious crashes in many series [16, 17, 31–33]. In Nigeria, Arosanyin et al. [18] found that, of the 344 commercial motorcyclists studied, 64% had driving license, 16% used helmet, 58.2% were aware of highway code, and 45% obeyed legal permissible passengers. Owoaje et al. [34] found zero rates of compliance with highway code. In Thailand, Siviroj et al. [35] found that, among 18,998 riders studied during Songkran festival, 44.2% had not been using helmet and 72.5% of passengers had not been using helmet. They also found that 75% agreed with the danger of nonhelmet use and 47.2% had been caught with the nonuse of a helmet before. They found that 83.7% had heard about road safety awareness campaign. Inasmuch as helmet use and safety laws are good, motorcycle rider education is also essential as this has been found to have positive effect on changing risky behavior of riders and motorcycle-related injuries in rural Thailand [36]. In Malaysia it was found that provision of exclusive lane for motorcycles reduced deaths by 60% [37].

Among our patients, the overall favorable functional outcome was 84.38% and mortality was 12.5%. In the study of 344 patients with traumatic brain injury in Nepal, Agrawal et al. [38] found good recovery in 87.7% and mortality of 4.5%. In Zaria, Nigeria, Muhammad [39] found mortality of 29% among the patients he studied. Hitimana et al. [40] found mortality of 13.2% among traumatic brain injury patients in their study. Among patients with mild head injuries in our study, favorable outcome was 94.34% and mortality was 3.77%. Jacobs et al. [41] studied 2784 patients with mild traumatic brain injuries and found favorable outcome in 87% and mortality was 2%. In our study, patients with moderate head injury had 95% favorable outcome and 5% mortality. In Hitimana et al. [40] the favorable outcome in moderate head injury was 75%, while, in mild head injury, it was 100%. Andriessen et al. [42] found that, in 169 moderate head injury patients in their study, the mortality was 17.68%. Among patients with severe head injuries in our study, the favorable outcome was 52.17%, while mortality was 39.13%. Andriessen et al. [42] found mortality of 41.79% among 335 severe head injury patients in their study. Boto et al. [43] found that, in 895 patients with severe head injury they studied, the mortality was 46.8%. In Hitimana et al. [40] study, the favorable outcome among severe head injury patients was 38.1%. Wang et al. [44] in Taiwan found 86.6% mortality among severe head injury patients. From above studies, our outcome was within what had been documented in many series.

In our study, intracranial lesion did not significantly affect the outcome. Rudehill et al. [45] studied 1,500 patients and found that intracranial lesions did not affect the outcome significantly. However, Chastain et al. [46] studied both CT scan and MRI (T2 and flair) findings of their patients and found that CT scan findings did not predict the outcome but MRI findings predicted the outcome. Our finding was in keeping with their CT scan finding. That was because our patients did CT scan only as there was no MRI in our center and in our city. Nelson et al. [47] in 890 CT scans of traumatic brain injury studied found that extradural hematoma positively predicted outcome. In our study, patients with extradural hematoma had 100% favorable outcome. In contrast, patients with subdural hematoma had 63.64% favorable outcome and mortality of 27.72%. In subdural hematoma, the impact is severe compared to extradural hematoma, and parenchymal injuries associated with subdural hematoma play major role in outcome determination. While we are heading towards zero mortality in acute extradural hematoma as predicted by Bricolo and Pasut [48], acute subdural hematoma outcome has not been encouraging. Wilberger Jr. et al. [49] found that, in 101 patients with acute subdural hematoma studied, the mortality was 66% and favorable outcome was 19%. Lobato et al. [50] found that, in 64 patients with extradural hematoma they studied, the favorable outcome was 68.8%, while the mortality was 28.1%. They noted that mortality was restricted to those in coma. Ayub et al. [51] in 108 patients with acute extradural hematoma they studied found that the favorable outcome was 69% and mortality was 8%. In 107 patients with extradural hematoma studied by Bricolo and Pasut [48], the favorable outcome was 89% and mortality was 5%. In combined study of acute extradural and acute subdural hematoma by Taussky et al. [52], mortality among patients with acute subdural hematoma was 41%, while those with acute extradural hematoma had 3% mortality. These studies with our study showed marked improvement in outcome in extradural hematoma when compared with acute subdural hematoma.

## 5. Conclusion

Our study found that almost all motorcycle riders were not using crash helmet. We also found that the outcome was significantly related to severity of injury at presentation. Patients with acute extradural hematoma had better outcome than those with acute subdural hematoma.

We believe that reasons advocated by opponents of helmet use were not strong enough to outweigh the protective effects of helmet. We thus recommend that the Federal Road Safety Corps (FRSC) in our country should broaden riders' education and enforce road safety regulations and helmet use laws. Government should consider providing exclusive lanes for motorcycles in our cities.

## Figures and Tables

**Table 1:** Age group frequency.

Age group	Frequency	Percent (%)
0–<10	3	3.13
10–<20	14	14.58
20–<30	24	25.00
30–<40	30	31.25
40–<50	17	17.71
50–<60	5	5.21
60–<70	3	3.13

Total	96	100

**Table 2:** Intracranial lesion versus Glasgow Outcome Score.

Intracranial lesions	Glasgow Outcome Score (GOS)					
	1 (%)	3 (%)	4 (%)	5 (%)	Total (%)	≥4 (%)
Extradural	0 (0.00)	0 (0.00)	0 (0.00)	4 (100)	4 (100)	4 (100)
Contusion	2 (8.00)	1 (4.00)	1 (4.00)	21 (84.00)	25 (100)	22 (88.00)
DAI	1 (12.5)	1 (12.5)	1 (12.5)	5 (62.5)	8 (100)	6 (75.00)
Edema	0 (0.00)	0 (0.00)	0 (0.00)	3 (100)	3 (100)	3 (100)
Subdural	3 (27.72)	1 (9.09)	1 (9.09)	6 (54.55)	11 (100)	7 (63.64)
Multiple	3 (16.67)	1 (5.56)	2 (11.11)	12 (66.67)	18 (100)	14 (77.78)

**Table 3:** Diagnosis versus GOS.

Diagnosis	Glasgow Outcome Score					
	1 (%)	3 (%)	4 (%)	5 (%)	Total (%)	≥4 (%)
Mild	2 (3.77)	1 (1.89)	6 (11.32)	44 (83.02)	53 (100)	50 (94.34)
Moderate	1 (5.00)	0 (0.00)	5 (25.00)	14 (70.00)	20 (100)	19 (95.00)
Severe	9 (39.13)	2 (8.70)	2 (8.70)	10 (43.48)	23 (100)	12 (52.17)

Total	12 (12.50)	3 (3.13)	13 (13.54)	68 (70.83)	96 (100)	81 (84.38)

*P* = 0.0002.
